# Anti-amyloid therapies for Alzheimer disease: finally, good news for patients

**DOI:** 10.1186/s13024-023-00637-0

**Published:** 2023-06-28

**Authors:** Vijay K. Ramanan, Gregory S. Day

**Affiliations:** 1 Department of Neurology, Mayo Clinic, Rochester, MN 55905 USA; 2 Department of Neurology, Mayo Clinic, 4500 San Pablo Road S, Jacksonville, FL 32224 USA

Recent advances in Alzheimer disease (AD) therapeutics represent a step in the right direction for patients with AD. In recent phase 3 trials for early symptomatic AD, lecanemab (CLARITY-AD) and donanemab (TRAILBLAZER-ALZ2) led to slowing of cognitive and functional decline, and positive alterations on disease-specific biomarkers [1, 2]. The substantial complexities of these new therapies highlight the frameshift required to ensure safe, effective use in clinical practice, and the unanswered questions that require new research to address.

Lecanemab and donanemab are humanized monoclonal antibodies targeting cerebral amyloid-beta (Aβ) plaques. Both medications are administered as weight-based intravenous infusions given every 2 (lecanemab) or 4 weeks (donanemab). Accumulation of Aβ is widely accepted to be an early marker of AD. Accordingly, there is great interest in understanding what happens *after* cerebral Aβ is lowered. Previous investigational agents targeting Aβ, including earlier anti-Aβ monoclonal antibodies, β-Site amyloid precursor protein cleaving enzymes (BACE inhibitors), and anti-Aβ vaccines [3], have demonstrated various levels of target engagement, with limited-to-no clinical benefits, and variable side effect profiles (Fig. 1). In comparison, lecanemab and donanemab display high potency for brain Aβ plaques, with most patients converting to amyloid-negative status by positron emission tomography (PET) by 12 months of therapy, and moderately favorable risk–benefit ratios in participants in recent phase 3 clinical trials [2, 4].

**Fig. 1:**
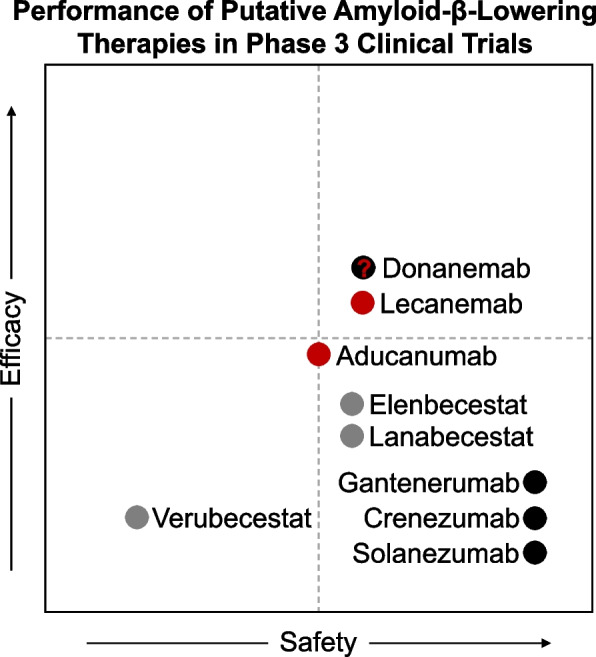
The relative safety and efficacy of putative amyloid-β-lowering therapies in patients with sporadic early-symptomatic Alzheimer disease. The efficacy and safety of selected amyloid-lowering therapies are depicted via scatter plot, referencing available data from Phase 3 clinical trials completed after 2016. Monoclonal antibodies accessible in the US under accelerated approval are depicted in red (donanemab remains under consideration). Other monoclonal antibodies are depicted in black. Agents with other mechanisms of action are depicted in gray. Dashed lines depict clinical meaningfulness. Agents in the top right quadrant met established endpoints for efficacy and safety. For more information consult clinicaltrials.gov, donanemab NCT04437511 [2] lecanemab NCT003887455 [1]; aducanumab NCT02477800, NCT02484547 [5]; gantenerumab NCT02051608, NCT01224106; solanezumab NCT01900665; crenezumab NCT03114657, NCT02670083; elenbecestat NCT012956486; lanabecestat NCT02972658, NCT02245737, NCT02783573; verubecestat NCT01953601

In the CLARITY-AD trial, participants receiving lecanemab experienced, on average, a ~ 25% slowing in cognitive decline over 18-months compared to those receiving placebo [1]. This equates to a 4-to-5-month delay in disease-related progression over the treatment period [6]. The treated group also displayed decreases in biomarkers of tau neuropathology, suggesting that Aβ-lowering may mediate other aspects of AD pathophysiology. Donanemab similarly met its primary outcome, with treatment slowing cognitive decline by an average of 35% compared to placebo [2]. Although full results of TRAILBLAZER-ALZ2 have yet to be released, aggregate data from these trials suggest that we are on the precipice of a new era of AD treatment and invites optimism concerning the potential that early initiation of Aβ-lowering therapies may slow disease progression. Still, the path ahead is perilous.

Infusion reactions (e.g., fever and flu-like symptoms, nausea/vomiting, skin reactions, and others) were reported in 25% of patients receiving lecanemab and 9% with donanemab [1, 2]. Although bothersome, the potential for more troubling side effects associated with anti-Aβ monoclonal antibodies, namely amyloid-related imaging abnormalities (ARIA), presents a much greater concern. ARIA can be subdivided into cases with cerebral edema (ARIA-E) or microhemorrhage/hemosiderosis (ARIA-H), both of which are more likely to occur early in the treatment course. Reported ARIA rates were slightly higher for donanemab (ARIA-E 24.0%, ARIA-H 31.4%) than lecanemab (ARIA-E 12.6%, ARIA-H 17.3%) in phase 3 trials [1, 2], although differences in study design limit direct comparisons. Over 90% of ARIA cases in the clinical trials were detected on planned safety MRIs in otherwise asymptomatic patients. Among patients with symptomatic ARIA, headache, visual disturbance, dizziness, or confusion were commonly reported [1, 2]. Serious adverse events with ARIA were observed in < 2% of patients treated with lecanemab [7]. There have even been patient deaths associated with lecanemab use within the CLARITY-AD open-label extension, most often in the context of exposure to anticoagulant therapies [8].

Overall, the findings from CLARITY-AD and TRAILBLAZER-ALZ2 emphasize that emerging therapies for AD will require judicious selection of appropriate patients for treatment (with the goal of maximizing benefit and minimizing harm), individualized counseling regarding potential risks and burdens of treatment, and robust pathways for monitoring safety and clinical response. Accurately discriminating patients with early symptomatic AD (i.e., mild cognitive impairment or mild dementia due to AD) in clinical practice is a nontrivial task, and biomarker confirmation (via cerebrospinal fluid tests or PET imaging) will be required to avoid exposing patients without amyloidosis to the risks of agents targeting Aβ plaques. Early findings indicate that ARIA risk is substantially elevated in *APOE* ε4 allele carriers (particularly among ε4/ε4 individuals), with guidelines for appropriate use recommending *APOE* genotyping in all patients considering lecanemab [7]. As a result, patients on treatment will need to have close clinical and MRI follow-up, as timely identification of ARIA is critical to determine whether treatment needs to be discontinued and whether goal-directed therapies (e.g., glucocorticoids, antiseizure medications) or hospitalization are required for management. Patients also need to be informed of the logistical burden associated with regular infusions/visits, the possibility of unanticipated financial costs, and the potential implications of other medical conditions including those which would otherwise require anticoagulant medications (considered a relative contraindication to anti-Aβ monoclonal antibody treatment due to the higher risk of symptomatic intracranial hemorrhage [7]). These recommendations and considerations increase the complexity of clinical decision making and emphasize the need for broad engagement of clinicians across multiple subspecialities.

Healthcare systems will need to adapt to this new framework. Barriers limiting access to PET imaging and cerebrospinal fluid biomarkers of AD will need to be addressed, requiring substantial investment in specialized neuroimaging infrastructure and insurance coverage (PET), and recruitment of skilled proceduralists to perform lumbar punctures. Clinical practices will also need to partner with or incorporate infusion centers, accommodate heightened volumes for clinical visits and MRIs, acquire expertise in detection and management of ARIA, and engage additional allied health professionals to aid in visit coordination and support patients on their treatment journeys.

Further research is also required to address several incompletely answered questions [5]. Discovery is needed for additional risk factors for ARIA, and relatedly, for factors that predict clinical response (or lack thereof). Unknowns exist around treatment effects in patients with clinically atypical AD, autosomal dominant AD, and AD with coexistent neurodegenerative pathologies. The optimal duration of therapy is also unclear. In CLARITY-AD, lecanemab treatment continued for 18 months. Donanemab treatment in TRAILBLAZER-ALZ2 used a different approach (treatment until amyloid PET negative), with 52% of treated individuals (71% of treated patients with intermediate burden of tau neuropathology confirmed on tau-PET) achieving amyloid clearance by 12 months [2]. Whether lecanemab treatment will delay or prevent cognitive impairment in cognitively normal individuals with elevated Aβ PET burden (i.e., preclinical AD) is being evaluated in the AHEAD 3–45 trial (clinicaltrials.gov: NCT04468659). Additional research is needed to identify the mechanisms and clinical meaning behind the association of treatment with lower brain volumes [9]. Further, there is a pressing need to increase diversity in AD clinical trials in order to narrow widespread disparities in care [10]. This backdrop highlights an opportunity for bedside and bench researchers at all stages to collaborate to better understand disease mechanisms, drug characteristics, patient needs, and points of intersection.

Importantly, drugs like lecanemab and donanemab are not cures for AD and there is no evidence to suggest that these agents halt or reverse cognitive impairment. Patients must be aware that these agents are appropriate for only a subset of individuals with AD. It is generally understood that Aβ is not the only factor in AD; treatments that target tau and other facets of the disease are needed to inform a precision-based combination approach to treatment of AD. Nevertheless, a field that has eagerly awaited good news is now poised to move forward at an accelerated pace.

## Data Availability

Not applicable.

## References

[1] van Dyck CH, Swanson CJ, Aisen P, Bateman RJ, Chen C, Gee M, et al. Lecanemab in Early Alzheimer's Disease. New England J Med. 2022. N Engl J Med . 2023;388(1):9–21. 10.1056/NEJMoa2212948. 10.1056/NEJMoa2212948 36449413

[2] Company ELa. Lilly's Donanemab significantly slowed cognitive and functional decline in phase 3 study of early alzheimer’s disease. 2023. Available from: https://investor.lilly.com/news-releases/news-release-details/lillys-donanemab-significantly-slowed-cognitive-and-functional. Accessed 20 May 2023.

[3] Panza F, Lozupone M, Logroscino G, Imbimbo BP (2019). A critical appraisal of amyloid-β-targeting therapies for Alzheimer disease. Nat Rev Neurol.

[4] McDade E, Cummings JL, Dhadda S, Swanson CJ, Reyderman L, Kanekiyo M (2022). Lecanemab in patients with early Alzheimer's disease: detailed results on biomarker, cognitive, and clinical effects from the randomized and open-label extension of the phase 2 proof-of-concept study. Alzheimer’s Res Therapy.

[5] Day GS, Scarmeas N, Dubinsky R, Coerver K, Mostacero A, West B (2022). Aducanumab Use in Symptomatic Alzheimer Disease Evidence in Focus: A Report of the AAN Guidelines Subcommittee. Neurology.

[6] Petersen RC, Aisen PS, Andrews JS, Atri A, Matthews BR, Rentz DM, et al. Expectations and clinical meaningfulness of randomized controlled trials. Alzheimer’s Dementia. 2023.Alzheimers Dement . 2023;19(6):2730–36. 10.1002/alz.12959. 10.1002/alz.12959 PMC11156248 36748826

[7] Cummings J, Apostolova L, Rabinovici GD, Atri A, Aisen P, Greenberg S, et al. Lecanemab: Appropriate Use Recommendations. J Prevent Alzheimer’s Disease. 2023. J Prev Alzheimers Dis. 2023;10(3):362–77. 10.14283/jpad.2023.30. 10.14283/jpad.2023.30 PMC10313141 37357276

[8] Reish NJ, Jamshidi P, Stamm B, Flanagan ME, Sugg E, Tang M (2023). Multiple Cerebral Hemorrhages in a Patient Receiving Lecanemab and Treated with t-PA for Stroke. N Engl J Med.

[9] Swanson CJ, Zhang Y, Dhadda S, Wang J, Kaplow J, Lai RYK (2021). A randomized, double-blind, phase 2b proof-of-concept clinical trial in early Alzheimer's disease with lecanemab, an anti-Aβ protofibril antibody. Alzheimer's Res Ther.

[10] Manly JJ, Gilmore-Bykovskyi A, Deters KD (2021). Inclusion of Underrepresented Groups in Preclinical Alzheimer Disease Trials-Opportunities Abound. JAMA Netw Open.

